# Tuberculous Soldiers on the Land

**Published:** 1919-08-23

**Authors:** 


### TUBERCULOUS SOLDIERS ON THE LAND.

The Inter-Departmental Committee on tuberculous soldiers have, it is now announced, made their report. At a time of acute congestion of national affairs such as the present, it is probably an extravagant hope that action on the lines recommended, always a late sequela to the reports of commissions, will soon be taken. All the same, to get something done in the matter is urgent and pressing enough. Discharged soldiers are the (certainly innocent) bugbear of many medical men, and of none more than the tuberculosis specialist. The lengthy treatment, often in institutions, that tuberculous patients need, accords very ill with the natural desire for home and for enjoyment, after the hardship of war, on the part of the consumptive soldier. And yet life at home, the ordinary home, means a pretty certain lapse of the disease into chronicity or worse, with risk of infection of the children of the household. What treatment in the sanatorium has mostly meant up to date has been, in numerous instances, friction and dismissal or self-discharge. The number of tuberculous soldiers is generally given as 35,000, perhaps a low estimate, seeing that pensions boards are steadily adding to the total as the result of re-examination of cases at first diagnosed as anæmia, bronchitis, or debility. In fine the appointment of a responsible committee of inquiry, especially charged with the provision of more adequate treatment, was plainly necessary. The personnel of this body, the reader may be reminded, consists of Major Astor, whose previous experience of tuberculosis higher administration is familiar, Sir Montagu Barlow, Sir Kingsley Wood, Colonel Raw, and Sir Owen Thomas, with representatives of the Pensions, Health, and Insurance Departments.

Their most notable recommendation is stated to be that the Government should establish eight or ten village settlements, where the convalescent tuberculous soldier can be looked after medically, while working at a trade and enjoying the company of his family. Presumably the trade will be one or other suitable to the many limitations of a consumptive's capacity: and indeed it is easier to think of unsuitable occupations, if only from consideration for the public health, than of suitable ones. In southern districts, as in Sussex for example, light work in the woodlands—the utilisation of undergrowth for faggots, hurdles, hop-poles and so on—has long been a staple industry. Fitter employment for consumptives with quiescent disease could hardly be got. Plate-laying on rural railway lines is another eminently favourable occupation. Consultation with labour authorities and with local employers should help in getting many of these poor men put in the way of a new start in life, useful not only to themselves but also to the national economy. For market gardening and allotment work, sure to figure in the shortish list of possible employments, will relieve the provision and transportation of food, which constitute now so great a burden upon our resources. Village settlements are also useful in encouraging de-urbanisation, which England as an industrial country needs so much. Last, but by no means least, satisfactory provision for men broken by the war will tend to allay the present acute discontent and unrest. All these considerations have doubtless been in the minds of those responsible for the recommendation.

And its carrying out, which cannot be a matter into which political motives enter, should be an early charge upon the national exchequer. This question of the disposal of tuberculous combatants has been a troubled one, so that to get it to some extent settled and done with should relieve the huge post-war agenda programme not a little. Colonial Governments, notably that of Queensland, have here displayed much more energy than have the home authorities, which is not in the proper order of things. Tuberculous soldiers are admittedly difficult patients, but the frequent phrase "grateful nation" is hardly applicable when their lot turns out to be no better than what it has been these last two or three years.

